# Relevance of mutations in protein deubiquitinases genes and *TP53* in corticotroph pituitary tumors

**DOI:** 10.3389/fendo.2024.1302667

**Published:** 2024-02-29

**Authors:** Monika Pękul, Magdalena Szczepaniak, Paulina Kober, Natalia Rusetska, Beata J. Mossakowska, Szymon Baluszek, Artur Kowalik, Maria Maksymowicz, Grzegorz Zieliński, Jacek Kunicki, Przemysław Witek, Mateusz Bujko

**Affiliations:** ^1^ Department of Cancer Pathomorphology, Maria Sklodowska-Curie National Research Institute of Oncology, Warsaw, Poland; ^2^ Department of Molecular Diagnostics, Holy Cross Cancer Center, Kielce, Poland; ^3^ Department of Molecular and Translational Oncology, Maria Sklodowska-Curie National Research Institute of Oncology, Warsaw, Poland; ^4^ Department of Experimental Immunology, Maria Sklodowska-Curie National Research Institute of Oncology, Warsaw, Poland; ^5^ Division of Medical Biology, Institute of Biology, Jan Kochanowski University, Kielce, Poland; ^6^ Department of Neurosurgery, Military Institute of Medicine - National Research Institute, Warsaw, Poland; ^7^ Department of Neurosurgery, Maria Sklodowska-Curie National Research Institute of Oncology, Warsaw, Poland; ^8^ Department of Internal Medicine, Endocrinology and Diabetes, Medical University of Warsaw, Warsaw, Poland

**Keywords:** corticotroph PitNET, Cushing’s disease, silent corticotroph tumor, *USP8*, *USP48*, *BRAF*, *TP53*

## Abstract

**Introduction:**

Corticotroph pituitary neuroendocrine tumors (PitNETs) develop from ACTH-producing cells. They commonly cause Cushing’s disease (CD), however, some remain clinically silent. Recurrent *USP8*, *USP48*, *BRAF* and *TP53* mutations occur in corticotroph PitNETs. The aim of our study was to determine frequency and relevance of these mutations in a possibly large series of corticotroph PitNETs.

**Methods:**

Study included 147 patients (100 CD and 47 silent tumors) that were screened for hot-spot mutations in *USP8*, *USP48* and *BRAF* with Sanger sequencing, while 128 of these patients were screened for *TP53* mutations with next generation sequencing and immunohistochemistry.

**Results:**

*USP8* mutations were found in 41% CD and 8,5% silent tumors, while *USP48* mutations were found in 6% CD patients only. Both were more prevalent in women. They were related to higher rate of biochemical remission, non-invasive tumor growth, its smaller size and densely granulated histology, suggesting that these mutation may be favorable clinical features. Multivariate survival analyses did not confirm possible prognostic value of mutation in protein deubiquitinases. No *BRAF* mutations were found. Four *TP53* mutations were identified (2 in CD, 2 in silent tumors) in tumors with size >10mm including 3 invasive ones. They were found in Crooke’s cell and sparsely granulated tumors. Tumors with missense *TP53* mutations had higher TP53 immunoreactivity score than wild-type tumors. Tumor with frameshift *TP53* variant had low protein expression. *TP53* mutation was a poor prognostic factor in CD according to uni- and multivariate survival analyses in spite of low mutations frequency.

**Conclusions:**

We confirmed high prevalence of *USP8* mutations and low incidence of *USP48* and *TP53* mutations. Changes in protein deubiquitinases genes appear to be favorable prognostic factors in CD. *TP53* mutations are rare, occur in both functioning and silent tumors and are related to poor clinical outcome in CD.

## 1 Introduction

Corticotroph neuroendocrine pituitary tumors (PitNETs) develop from normal corticotroph cells of pituitary gland i.e. the cells that are characterized by production of adrenocorticotropic hormone (ACTH) and expression of lineage-specific transcription factor TPIT. Corticotroph PitNETs commonly cause Cushing’s disease (CD) which is a set of clinical symptoms caused by cortisol excess. However, approximately 20% of tumors originated from corticotroph pituitary cells are endocrinologically non-functioning. These silent tumors (commonly referred to as silent corticotroph adenomas, SCAs) do not affect hormonal balance and do not cause CD. Unlike people with CD, patients with SCAs suffer from neurological symptoms (for example visual impairment) caused by tumor mass, but do not experience hypercortisolism. In general, corticotroph origin of these tumors is determined following histopathological examination based on positive TPIT immunohistochemical staining (1).

Therapy for pituitary corticotroph PitNETs includes transsphenoidal surgery, medical treatment and/or radiation therapy. Transsphenoidal surgery is the recommended treatment of choice for all ACTH-producing tumors. In CD patients biochemical remission rates range from 65–98%, with higher rates when the tumor is identified on MRI and completely removed. Medical therapy is necessary for persistent or recurrent disease after transsphenoidal surgery or if surgery is contraindicated. The available pharmacotherapy approaches are directed at three targets: ACTH production by a corticotroph tumor, steroidogenesis in the adrenal gland, and glucocorticoid receptors. Radiation therapy is used when pituitary surgery is unsuccessful as the last option for aggressive tumors or poor surgical candidates (2).

In the last few years a notable progress in the understanding of molecular background of corticotroph pituitary tumors has been made (3). The studies focused on somatic mutations in corticotroph tumors revealed recurrent mutations in *USP8* gene (encoding deubiquitinase protein) in a notable proportion of patients (4–6) as well as some other, much less frequent mutations in *USP48* (7, 8), *BRAF* (8) and *TP53* genes (7) that were found in *USP8* wild type tumors. *USP8* mutations appear to determine tumors molecular profile (9, 10) and response to particular pharmacological treatment (11–13). The aim of this study was to determine the frequency of these mutations in collection of corticotroph tumors tissue from a large cohort of patients and to determine their clinical significance.

## 2 Materials and methods

### 2.1 Patients’ characteristics

The study included 147 patients with corticotroph tumors: 100 patients with CD and 47 patients with silent corticotroph PitNETs. All patients were treated with surgery at the Department of Neurosurgery, Maria Sklodowska-Curie National Research Institute of Oncology in Warsaw and Department of Neurosurgery, Military Institute of Medicine - National Research Institute in Warsaw, between years 2004-2020 (CD patients, with only 13 patients treated before 2012) and 2010-2020 (SCA patients). CD patients had evident clinical signs and symptoms of hypercortisolism verified according to biochemical criteria: increased urinary free cortisol (UFC) in three 24h urine collections; disturbed cortisol circadian rhythm (with midnight serum cortisol level or late night salivary cortisol) no suppression of serum cortisol levels to <1.8 µg/dL after an overnight dexamethasone suppression test (1 mg at midnight).

The pituitary etiology of Cushing`s syndrome was confirmed based on the measurement of morning serum ACTH level (and additionally by positive result of a corticotropin-releasing hormone stimulation test in patients with normal ACTH level (100 mg i.v.) as well as magnetic resonance imaging of pituitary. In patients with inconclusive MRI bilateral inferior petrosal sinus sampling (BIPSS) was applied.

The group of CD patients included 4 patients aged below 18 years (patients aged 13, 15, 16 and 17 years), while the remaining patients were adults. Patients with silent tumors were diagnosed as cases of the nonfunctioning pituitary tumors. The endocrinological work up showed neither signs nor symptoms of hypercortisolemia, no history of exogenous glucocorticosteroid treatment. The hypercortisolemia was excluded and normal cortisol circadian rhythm was assessed with one or two screening tests (increased UFC in three 24h urine collections, no suppression in the late night salivary cortisol or no suppression of serum cortisol levels after an overnight 1 mg dexamethasone suppression test). They all had MRI-confirmed pituitary tumor and were qualified for pituitary surgery due to symptoms of mass effect, visual disturbances or neurological deficits.

ACTH levels were assessed using IRMA (ELSA-ACTH, CIS Bio International, Gif-sur-Yvette Cedex, France) with analytical sensitivity of 2 pg/mL (reference range: 10–60 pg/mL). Serum cortisol concentrations were determined by the Elecsys 2010 electrochemiluminescence immunoassay (Roche Diagnostics, Mannheim, Germany) with sensitivity of the assay of 0.02 μg/dL (reference range: 6.2–19.4 μg/dL). UFC was determined after extraction (liquid/liquid with dichloromethane) by electrochemiluminescence immunoassay (Elecsys 2010, Roche Diagnostics, Mannheim, Germany) - reference range: 4.3-176 μg/24h. All the functioning tumors and SCA samples were ACTH-positive in immunohistochemical staining against pituitary hormones (ACTH, GH, PRL, TSH, FSH, LH, α-subunit) and had characteristic ultrastructural features of corticotroph tumors as determined with electron microscopy. SCA samples were subjected to additional immunostaining against TPIT to verify the corticotroph origin of the tumors. The tumors were classified as sparsely or densely granulated with the use of electron microscopy.

Invasive tumor growth status was assessed based on MR imaging using Knosp’s scale (14). Tumors with Knosp grades 0, 1 and 2 were considered non-invasive, whereas tumors with Knosp grades 3 and 4 were considered invasive.

Biochemical remission after surgery in CD patients was defined as morning serum cortisol level lower that 1.8 µg/dL at first, second and/or the third postoperative day.

Overall survival was defined as time from surgery to patient’s death or to the last follow-up data collection (for alive patients). For recurrent tumors the date of the first surgery was used for the analysis. The information on survival status was obtained from Polish National Cancer Registry and were actualized on 18.01.2023. Additionally, the information on the cause of death was obtained by contacting family members.

Patient’s characteristics are presented in **Table 1**. The study was approved by the local Ethics Committee of Maria Sklodowska-Curie National Research Institute of Oncology, Warsaw, Poland. Each patient provided informed consent for the use of tissue samples for scientific purposes.

**Table 1:** Summary of clinical features of patients with Cushing’s disease and silent corticotroph tumors.

Clinical Feature	Cushing’s disease	Silent corticotroph PitNETs
**Number of patients**	n=100	n=47
**Sex** females males	79 21	20 27
**Age at surgery** (years; median (range))	44 (13-78)	55 (19-77)
**Cortisol 08:00 h** (µg/dL; median (range); reference range: 6.2-19.4 µg/dL)	24.4 (10.8 – 73.4)	13.7 (3.1 – 29.7)**
**ACTH 08:00 h** (pg/dL; median (range); (reference range: 10-60 pg/mL)	74.6 (19.6 – 390)	44.4 (10.1 – 74.9) ***
**UFC** (μg/24 h; median (range); (reference range: 4.3-176 µg/24h)	483.5 (26 – 830)	94.7 (13.7 - 139)****
**Tumor largest size** (mm; median (range))	11.5 (3 - 62)	24 (15 – 62)
**Invasive tumor growth** Non-invasive (Knosp grade 0, 1, 2) invasive (Knosp grade 3, 4)	73 27	28 19
Pathomorphology* sparsely granulated densely granulated Crooke’s cell	29 66 2	17 27 3

*not available for 3 CD patients; **not available for 4 patients with silent tumors; ***not available for 17 patients with silent tumors; ****not available for 26 patients with silent tumors.

This study group is partially composed of the patients who were already included in our previous research including 70 patients with CD and 25 patients with SCAs (9, 15, 16).

### 2.2 Sanger sequencing

Genomic DNA was isolated from formalin-fixed and paraffin-embedded (FFPE) tumor tissue sections using RecoverAll™ Total Nucleic Acid Isolation Kit for FFPE [Thermo Fisher Scientific, Waltham, Massachusetts, USA], measured using NanoDrop 2000 [Thermo Fisher Scientific, Waltham, Massachusetts, USA] and stored at -70°C.

Hot spot regions of *USP8* (coding for 14-3-3 binding motif, chr15:50,490,362-50,490,596; hg38), *USP48* (chr1:21,729,673-21,729,899; hg38) and *BRAF* (chr7:140,753,278-140,753,450; hg38) were analyzed in tumor samples from 147 patients with Sanger sequencing. DNA was PCR-amplified with FastStart Taq DNA Polymerase (Roche Diagnostics, Mannheim, Germany) using GeneAmp 9700 PCR system (Applied Biosystems, Foster City, CA, USA). PCR product was purified using ExoStar (GE Healthcare Life Sciences, Pittsburgh, PA, USA), labeled with BigDye Terminator v.3.1 (Applied Biosytems, Foster City, CA, USA) according to the manufacturer’s instructions and analyzed by capillary electrophoresis with the ABI PRISM 3300 Genetic Analyzer (Applied Biosystems, Foster City, CA, USA). The following nucleotide/protein sequences were used as reference: NM_005154.5/NP_005145.3; NM_032236.8/NP_115612.4 and NM_004333.6/NP_004324 for detecting variant in *USP8*, *USP48* and *BRAF*, respectively.

### 2.3 *TP53* sequence analysis with targeted next generation sequencing (NGS)

The *TP53* gene was sequenced by NGS using Ion Torrent technology. For this purpose, a pre-designed panel Ion AmpliSeq TP53 Panel (TP53.20140108, Ion AmpliSeq Community Panel) covering the entire protein-coding sequence of *TP53* and exon-intron boundaries was selected by the online tool Ion Ampliseq Designer (Thermo Fisher Scientific).

Sample concentrations were measured using Qubit 2.0 Fluorometer (Life Technologies) with the dsDNA HS Assay Kit. Next, according to the manufacturer’s instructions, gene libraries were prepared using Ion AmpliSeq™ Library Kit Plus (Thermo Fisher Scientific) and the primers. Sequencing was performed using Ion GeneStudio™ S5 Prime System (Thermo Fisher Scientific).

The designed panel detects single nucleotide polymorphisms, as well as deletions and insertions of up to 20 nucleotides. The method’s limit of detection is ≥ 5% at the 99% confidence level. Detection of homopolymeric variants is also limited due to specificity of Ion Torrent technology.

To analyze the results generated, free tools available online wAnnovar (https://wannovar.wglab.org/) and Sheshat (https://p53.fr/tp53-database/seshat) were used to describe the obtained variants. For the analysis, Integrative Genomics Viewer (https://igv.org/) was also used to provide a visual representation of the detected alterations. The filtered variants were then checked using the Clinvar (https://www.ncbi.nlm.nih.gov/clinvar/) and the UMD TP53 (https://p53.fr/download-the-database) dedicated to the *TP53* gene database, as well as the Varsome bioinformatics tool (https://varsome.com/). Classification of mutations was based mainly on the interpretation of their significance provided by Clinvar and UMD TP53 (https://p53.fr/the-database), and if a particular alteration was not classified, the Varsome tool was used to determine pathogenicity according to ACMG guidelines.

The identified mutations were confirmed with Sanger sequencing.

### 2.4 Immunohistochemistry

Immunohistochemical staining (IHC) was applied to visualize TP53 expression level in 112 tumors that were included in *TP53* coding sequence analysis. Envision Detection System (DAKO, Glostrup, Denmark) was used for staining procedure according to manufacturer’s recommendations. Four-μm FFPE tissue sections were deparaffinized with xylene and rehydrated in a series of ethanol solutions of decreasing concentration. Heat-induced epitope retrieval was achieved by 30 minutes incubations of the samples in Target Retrieval Solution pH 9 (DAKO) in a 96°C water bath. The slides were treated with a Blocker of Endogenous Peroxidase (DAKO) for 5 minutes followed by incubation with the primary monoclonal mouse anti-human TP53 antibody (clone DO-7, ready to use concentration, DAKO) for 1h. Diaminobenzidine tetrahydrochloride (Dako) was used as substrate to visualize immunoreactivity followed by hematoxylin nuclear counterstaining. Analysis of nuclear immunohistochemical reactivity was performed by calculating H-score, that combines information on both reaction intensity (scored from 0 to 3) and number of the cells with a given intensity. Previously reported formula was used for quantification (17). Three high power fields (magnification x400) were evaluated for each sample. Scoring results were analyzed as continuous variables.

### 2.5 Statistical analysis

Two-sided Mann–Whitney U-test was used for analysis of continuous variables in comparison of two groups. Kruskal-Wallis test with *post hoc* analysis with Dunn’s test and False Discovery Rate correction for multiple testing was applied for comparisons of more than two groups. Fisher’s exact test was applied for the analysis of proportions. Significance threshold of α = 0.05 was adopted. Data was analyzed using GraphPad Prism 6.07 (GraphPad Software).

Analysis of patient survival was carried out in R (v4.2.2), utilizing survival package. Proportional hazard Cox model was utilized for modelling patient survival (CD-related deaths). Wald test was applied for the hypothesis testing. Uni- and multivariate models were built.

## 3 Results

### 3.1 The prevalence of *USP8*, *USP48*, *BRAF* and *TP53* mutations in corticotroph PitNETs

Mutation status of hot-spot regions in *USP8*, *USP48* and *BRAF* was determined in 100 functioning corticotroph tumors from the patients suffering from CD and in 47 silent corticotroph tumors from patients without evidence of Cushing’s syndrome.

*USP8* mutations were identified in 45/147 (30.6%) of all corticotroph tumors that were analyzed. They occurred mainly in functioning CD-related PitNETs (41/100; 41%) and only in 4/47 (8.5%) of silent tumors (9). The identified variants were p.P720R (found in 22 patients), p.S718del (n=14), p.S718P (n=7) and p.P720Q (n=2).

*USP48* hot-spot mutations were found in 6/100 (6%) tumors from CD patients, while no variants in this gene were identified in silent tumors. Five *USP48* mutations were missense variants p.M415V, one was p.M415I. They all were identified in patients without *USP8* mutation. We did not find hot spot *BRAF* mutations in any of the patients.

One hundred and twenty-eight corticotroph tumors (81 CD-related and 47 silent PitNETs) were included in *TP53* mutations screening. Four mutations (3 missense variants and one frameshift mutation) were identified in 2/81 (2.5%) CD-related functioning tumors and in 2/47 (4.2%) silent corticotroph PitNETs. Thus, the mutations were identified in a total of 4/128 (3.1%) patients. All *TP53*-mutated patients were negative for *USP8* or *USP48* mutations indicating that all these mutations are mutually exclusive. Detailed description of patients with *TP53* mutations is provided below.

The characteristics of the mutations found in the analyzed genes are summarized in **Supplementary Table 1**.

### 3.2 Relevance of the mutations in protein deubiquitinases

Basic demographical features such as patients’ sex and age as well as clinical parameters including results of biochemical tests and tumor characteristics were compared between patients without mutations in deubiquitinating enzymes and *USP8*-mutated and *USP48*-mutated patients. Patients with CD and those suffering from SCA represent clinically separate groups and the mutations occur basically in functioning corticotroph PitNETs, hence, this analysis is focused on CD patients.

**Figure 1:**
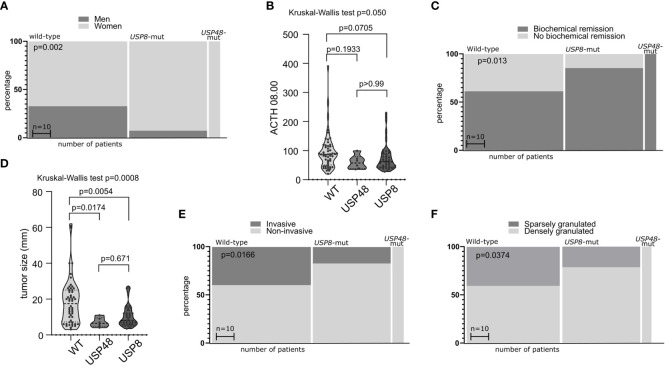
The relationship between mutations in deubiquitinase genes and demographical/clinical characteristics of patients with Cushing’s disease. **(A)** Difference in proportion of male and female patients in mutated and wild-type patients. **(B)** Difference in morning plasma ACTH level in patients stratified according to *USP8* and *USP48* mutation status. **(C)** Difference in the proportions of patients with biochemical remission after surgery in patients grouped according to mutational status. **(D)** Comparison of the size of tumors with and without mutations in proteins of deubiquitinases genes. **(E)** Difference in proportion of invasive and non-invasive corticotroph PitNETs between tumors with mutation and wild-type tumors. **(F)** Difference in the proportion of sparsely granulated and densely granulated tumors.

We observed an evident difference in the ratio of male/female patients between the groups of CD patients stratified according to *USP8* mutation status (p = <0.0001) (**Figure 1A**). Wild-type patients included 32 men (32 out of 53 patients in this group, 60.4%) whereas only 3 male patients were among *USP8*-mutated patients (3 out of 41 patients in this group, 7.3%). All *USP48-*mutated patients were women. No significant difference was observed in patients’ age. Difference in morning plasma ACTH level was observed in comparison of three groups of patients (*USP8*-mutated, *USP48*-mutated and wild type) with Kruskal Wallis test (p = 0.050), however, the observed difference between wild-type patients and *USP8*-mutated tumors did not cross significance threshold in *post hoc* analysis with p-adjustment (mean 95.1 vs. 60.27 pg/dL; p= 0.0705). No difference was seen in *USP48*-mutated tumors as compared to those without mutations in protein deubiquitinases genes (**Figure 1B**).

No significant difference was observed in levels of cortisol: morning and midnight plasma cortisol levels and 24h UFC. Importantly, difference in biochemical remission rate after surgery was observed in patients with *USP8* mutations, *USP48* mutations and wild type patients (p = 0.0079) (**Figure 1C**). Biochemical remission was observed in 32/53 (60.38%) of wild-type patients, in 35/41 (85.36%) *USP8*-mutated patients and in all *USP48*-mutated patients.

Distinct characteristics of mutated and wild type corticotroph tumors were also observed including difference in the size (largest tumor size) of tumors of three groups (p = 0.0008). Both *USP8* and *USP48* tumors were smaller than wild type tumors (mean 10.56 vs 18.55 mm; p = 0.0054 and mean 7 vs 18.55 mm; p = 0.0174, respectively) with no difference between *USP8* and *USP48*-mutated adenomas (**Figure 1D**) according to *post hoc* analysis.

Different rate of invasive tumors was found in *USP8*-mutated, *USP48*-mutated and wild type functioning tumors (p = 0.0166) (**Figure 1E**). Group of *USP8*-mutated PitNETs included 7 invasive tumors (7 out of 41 tumors in this group; 17.07%), tumors with *USP48* mutations were all non-invasive, while 21 out of 53 wild-type tumors (39.62%) were invasive. We also observed significantly different proportion of sparsely vs densely granulated tumors (p = 0.0374) (**Figure 1F**). Only 9/41 (21.2%) *USP8*-mutated tumors were sparsely granulated, all tumors with *USP48* mutation were densely granulated (excluding one patient for whom tumor granulation pattern was not assessed), while 20/49 (40.82%) wild-type tumors were sparsely granulated (granulation status for one wild-type patient was unavailable). Two Crooke’s cell adenomas were not included in this analysis and both these tumors were *USP8*/*USP48* wild type. Results are summarized in **Table 2**.

**Table 2:** The comparison of demographical and clinicopathological characteristics in patients with Cushing’s disease stratified according to *USP8* and *USP48* mutation status.

Patients feature	*USP8*/*USP48* wild type (n=53)	*USP8*-mutated (n=41)	*USP48*-mutated (n=6)	p-value	
Sex number of males (percentage)	32 (60.4%)	3 (7.3%)	0	**p<0.002**	Fisher’s exact test
Age (years) mean (range)	47.04 (13-78)	43.49 (16-74)	41.67 (33-59)	p = 0.4308	Kruskal-Wallis test
ACTH (pg/dL) mean (range)	95.10 (19.6-390)	74.51 (27.9-230)	60.27 (35.6-99.5)	**p = 0.05**	Kruskal-Wallis test
Cortisol 8:00 (µg/dL) mean (range)	25 (11.61-73.4)	26.24 (10.8-49.7)	21.82 (11.54-30.37)	p = 0.3543	Kruskal-Wallis test
24h UFC (μg/24 h) mean (range)	486.8 (26-1230)	493 (101.9-1060)	451.1 (122.7-961.8)	p = 0.9289	Kruskal-Wallis test
Biochemical remission number of patients with remission (percentage)	32 (58.5%)	35 (85.4%)	6 (100%)	**p = 0.013**	Fisher’s exact test
Tumor largest size (mm) mean (range)	18.55 (3-62)	10.56 (3.5- 27)	7 (4-11)	**p = 0.0008**	Kruskal-Wallis test
Invasive growth number of invasive tumors (percentage)	21 (39.6)	7 (17%)	0	**p = 0.0166**	Fisher’s exact test
Granulation pattern* number of sparsely granulated tumors (percentage)	20 (40.82%)	9 (21.2%)	0	**p = 0.0374**	Fisher’s exact test

*Not assessed for 3 patients; Crooke’s cell adenomas not included. p-values in bold denote statistically significant result.

### 3.3 Role of *TP53* mutations and TP53 immunoreactivity in corticotroph PitNETs.

Only 4 patients with mutations in *TP53* coding sequence were found in the entire patients cohort. These identified variants included 3 missense mutations c.817C>T p.Arg273Cys; c.542G>A p.Arg181His and c.332T>C p.Leu111Pro. These mutations are reported in UMD TP53, where the first one is classified as “pathogenic”, while the remaining two missense mutations are classified as “possibly pathogenic”. One frameshift mutation p.Lys382AsnfsTer40 was identified in C-terminal regulatory domain. The position of p.Lys382 is a site of TP53 regulatory acetylation (18). This mutation is reported in UMD TP53 database as probably pathogenic.

Two *TP53* mutations were found in functioning corticotroph tumors and two were identified in silent tumors. All *TP53* mutations were found in macroadenomas including 3 invasive (Knosp grade 4 in one patient and grade 3 in two patients) and one non-invasive tumor (Knosp grade 2). Two *TP53*-mutated CD patients were 58-year-old man and 78 year-old woman. Both were deceased with overall survival of 40 and 63 months, respectively. In turn, two patients with *TP53*-mutated silent PitNETs were 40-year-old woman and 41 year-old man, both are alive (with follow up of 129 and 73 months, respectively). Medical data records of these two patients did not include any information suggesting a possible genetic burden predisposing to early cancer onset due to germline *TP53* mutation. Interestingly, *TP53* mutations were found in 2/5 Crooke’s cell tumors and the other two were SG tumors.

**Figure 2:**
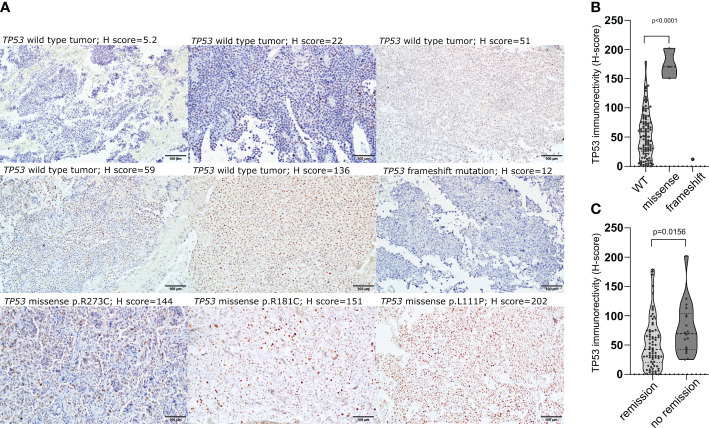
The relationship between *TP53* coding sequence mutation and TP53 expression in corticotroph PitNETs as well as relevance of protein expression. **(A)** Immunohistochemical staining with antibody against TP53 (clone DO-7) in *TP53*-mutated tumors and examples of immunostaining in *TP53* wild-type tumors; **(B)** Difference in TP53 protein expression in tumors with *TP53* missense mutations, frameshift mutation and *TP53*-wild-type tumors; **(C)** Difference in *TP53* expression in CD patients with and without biochemical remission after surgery.

The expression of TP53 was assessed in 112 patients with immunohistochemistry and the immunoreactivity was assessed with semiquantitative approach. Vast majority of tumors (92/112; 82.1%) presented low TP53 expression (H-score<100), while 2 samples were considered to be negative (less than 1% of positive cells). Moderate expression (H-score between 100 and 200) was observed in 17/112 (15.2%) tumors, while high expression (H-score >200) was found in 1 tumor. All 3 tumors with missense *TP53* mutations had moderate/high immuno-score (H-score of 150.8; 170.3; 202.1) and TP53 immunoreactivity was significantly higher in these tumors than in wild-type ones (mean H-score 174.4 vs 55.63, p<0.0001), whereas very low TP53 immunoreactivity (H-score of 12) was observed in the tumor with frameshift mutation (**Figures 2A, B**). CD patients with biochemical remission after surgery had lower TP53 immunoreactivity (mean H-score 48.24 vs 78.57, p = 0.0068) (**Figure 2C**).

No difference between functioning and silent tumors was observed as well as between SG, DG and Crooke’s cell tumors. No correlation was found between cortisol, ACTH, UFC levels and TP53 immunoreactivity (H-score) (neither in the analysis of the entire group nor in CD patients only). No difference in TP53 expression was observed between patients with *USP8*, *USP48* mutations and patients without mutations in deubiquitinase genes. Similarly, no difference between invasive and non-invasive tumors and tumors grouped according to Knosp grade was found. We did not find relationship between patients’ survival and TP53 immunoreactivity treated either as continuous variable (H-score) or categorical variable (low vs moderate/high expression).

### 3.4 Evaluation of prognostic role of the recurrent *USP8*, *USP48* and *TP53* mutations in Cushing’s disease.

The comparison of the clinical characteristics in *USP8*, *USP48* and *TP53*-mutated groups of the patients indicate its possible relation with outcome. Using patients overall survival data with median follow-up of 76.5 months (ranging from 34 to 227 months) we assessed whether these mutations have possible prognostic value. In our group of CD patients there were 14 deceased patients according to information from Polish National Cancer Registry (dated 18.01.2023). The cause of patients death was determined by contacting with patients family members. Based on the obtained information, the follow up data was corrected. Death of five patients was considered as not related directly to CD (COVID-19 in 4 patients and uterine cancer in 1 patient). The observations for these patients were treated as censored in the CD-related deaths analysis.

**Figure 3:**
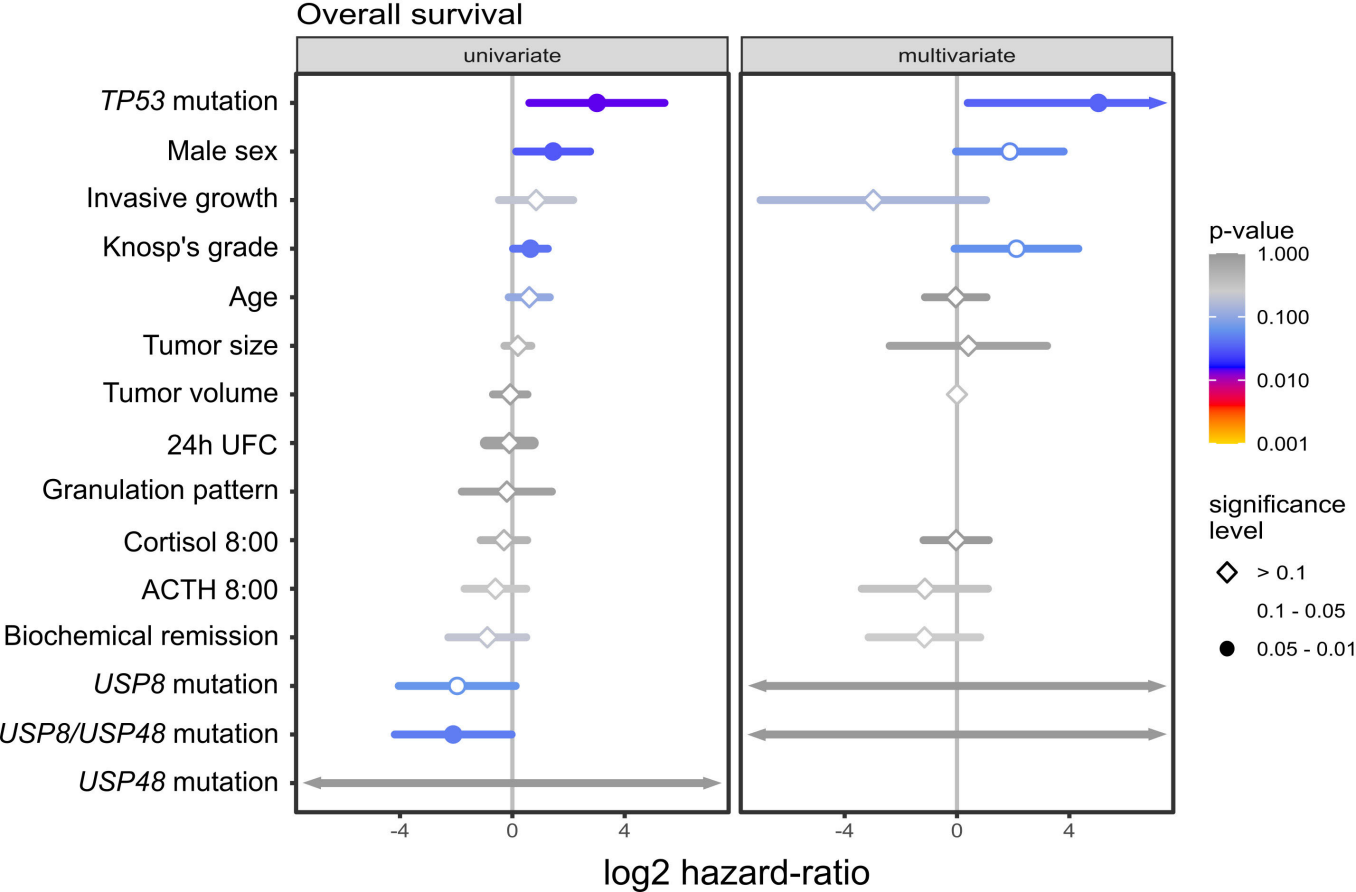
The results of univariate and multivariate survival analysis of selected demographical and clinical features including *USP8*, *USP48* and *TP53* mutations in patients with Cushing’s disease.

In univariate survival analysis out of 15 included demographical/clinical features 4 parameters showed statistical significance, as shown in **Figure 3**. *TP53* mutations, male gender and higher Knosp’s grade had higher risk of death while presence of *USP8* or *USP48* mutations (considered as a single parameter) was associated with the reduced risk. In multivariate analysis only *TP53* mutations displayed statistical significance, being associated with higher risk of death. We observed a trend of increased risk of death in this model, associated with male gender and Knosp’s grade, which was of borderline statistical significance (p-value of 0.0544 and 0.0598, respectively). In multivariate model statistical significance of *USP8*, *USP48* mutations was not observed. This may be explained by other associated features being more predictive of survival; specifically, as observed in our study, *USP8* and *USP48* mutations are both associated with female sex and we observe trend towards worse survival in males. Results are presented in **Figure 3**.

## 4 Discussion

DNA sequencing studies in corticotroph PitNETs revealed a few recurrent mutations that play a role in pathogenesis of these tumors. The identified genetic changes include point mutations in genes coding for protein deubiquitinases *USP8* (4–6) and *USP48* (7, 8) as well as in known cancer-related genes *TP53* (7) and *BRAF* (8). Our study was to validate the frequency of these mutations and their clinical relevance in a possibly large cohort of patients with corticotroph tumors (9, 15, 16).

In our study, changes in *USP8* were the most frequent mutations, occurring mainly in functioning corticotroph PitNETs. They were present in 41% of tumors causing CD, which is comparable to previously reported prevalence of *USP8* variants in different CD patients’ populations (4, 6, 12, 19, 20). However, both lower (21–24) and higher (5) proportions of *USP8*-mutated CD patients were also reported. Mutations in *USP48* and *TP53* were much less common and were mutually exclusive. Each of them was also mutually exclusive with *USP8* mutations, as was also reported (7). Mutations in deubiquitinases genes were found mainly in functioning corticotroph tumors with only 4 *USP8* mutations in SCAs (these patients were already described in details (9)). *TP53* mutation occurred in both functioning and clinically silent tumors. We did not detect any *BRAF* mutation in our patient’s cohort, in contrary to results from NGS-based study (8). Lack of *BRAF* mutations in corticotroph PitNETs was also described by other authors (19, 25).

Demographical and clinical associations of *USP8* mutations in patients suffering from CD were examined in a few studies (4, 6, 12, 19, 22, 23, 26–29). Most of these observations are confirmed by our results. Changes in *USP8* hot spot region were identified predominantly in female patients as previously (4, 5, 23, 27, 30). We found these mutations to be related to biochemical remission after surgery as also described previously (4, 12, 27). In our study group CD patients with biochemical remission had significantly smaller, commonly noninvasive tumors than patients who did not achieve remission (data not shown). It can be presumed that tumors with mutations in *USP8* grow less aggressively than wild type ones because of difference in tumor biology and the difference in the remission rates indirectly suggest the probable favorable value of *USP8* mutations. Accordingly, our results showed significantly lower tumor size and lower proportion of invasive tumors among *USP8*-mutated ones that confirms previous observation (5) and indicates their less invasive nature as compared to wild-type tumors. Additionally, in our study majority of *USP8*-mutated CD-causing tumors were densely granulated and this histological subtype is related to better clinical outcome as compared to SG corticotroph PitNETs (31).

Recent *in vitro* studies showed that changes in *USP8* are related to better response to somatostatin analog – pasireotide (11, 13). The use of this drug is currently recommended in patients with persistent CD or inoperable functioning corticotroph tumors. Unfortunately, since low proportion of our patients received adjuvant treatment due to lack of remission and the treatment was not homogenous and included both medical therapy and radiotherapy, the analysis of the role of *USP8* in pasireotide response in our group was not feasible.

Interestingly, the demographical/clinical profile of *USP48*-mutated patients resemble the one of *USP8*-mutated individuals. We found *USP48* mutation only in female patients, with lower ACTH level as compared to wild-type patients, who all achieved biochemical remission after surgery. *USP48*-mutated tumors were smaller than wild-type tumors and they all were non-invasive. It indicates that these mutations, similarly to changes in *USP8*, are related to favorable clinical characteristics, as also previously observed (7). Of note, *USP8*- and *USP48*-mutated tumors seem to share at least some molecular features such as low p27 expression (32) or high *POMC* expression (8). We also have to point out a discrepancy concerning *USP48* mutations that were found only in non-invasive tumors in our study but were observed in invasive corticotroph PitNETs in study by Abraham et al. (19).

We made an attempt to determine the possible prognostic role of the mutations in protein deubiquitinases genes with survival analysis. In our univariate analysis we observed the relation of the incidence of *USP8*/*USP48* mutation (considered as single parameter) with better survival, but the lower risk in mutated patients was not observed in multivariate analysis. We cautiously interpret the results of univariate analysis. Importantly, in our study male gender was a better predictor of death in multivariate survival analysis (bordering on statistical significance) than deubiquitinase mutation. Differences in female and male CD patients were described previously (33) with tendency toward worse outcome in male patients observed in some of the studies (34, 35). According to our observations both *USP8* and *USP48* mutations occur almost exclusively in female patients. These data are covariant, hence, there is still a question whether *USP8/USP48* mutations could be positive prognostic factor that occurs more often in women or whether male patients have worse prognosis due to other unclear factors. Therefore, we cannot exclude the simple possibility that the role of *USP8*/*USP48* mutations observed in univariate analysis reflects the fact that mutated patients are mainly women who clearly tend to have better prognosis than men. Therefore, in spite of the observed relationship between *USP8*/*USP48* mutations and favorable prognostic features we were not able to unequivocally confirm that *USP8*/*USP48* mutation itself has a direct prognostic value. Discovery of *TP53* mutations in a notable proportion of corticotroph PitNETs negative for *USP8* mutations (7) was somehow surprising, since the role of *TP53* in pituitary tumors was a matter of previous research (36–41). Former mutational screening of *TP53* DNA sequence (exons 4-9) with single-strand conformation polymorphism technique (SSCP) didn’t reveal mutations in corticotroph tumors (36). Immunoreactivity against TP53, which was commonly considered as equivalent to mutational screening (37) was used as one of the criteria of atypical pituitary adenomas until 2017. The published results on its clinical significance were inconclusive. Some reports suggested a prognostic role of TP53 immunoreactivity in corticotroph tumors (38, 39) but other failed to confirm this value (40, 41).

Very recent studies, that followed discovery of *TP53* mutations in exome-seq results of *USP8*-wt tumors (7) confirmed their incidence in corticotroph PitNETs. The mutations were found in 9/86 (42) and 5/22 (43) patients that were included in sequencing of *TP53* coding regions or exome-seq, respectively. Our results show that mutations in this gene occur in corticotroph PitNETs but with lower frequency in our cohort than in previously reported groups of patients. We found *TP53* mutations in 3.3% of analyzed corticotroph PitNETs, which is notably less than 10.5% reported by Perez−Rivas LG et al. (42) and 22.7% described by Uzilov et al. (43). Difference in the frequency of *TP53* mutations probably reflects differences between study groups. Tumors examined by Uzilov et al. (43) were intentionally enriched for aggressive tumors, while in our study we included possibly large retrospective cohort of histologically confirmed corticotroph tumors without any preselection of patients. In turn, study by Perez−Rivas LG et al. (42) included patients with functioning corticotroph tumors only, but with lower proportion of *USP8*-mutated patients than included in our study (29% vs 41% *USP8*-mutated functioning tumors in our study group). We suspect that different proportion of *USP8*-mutated and wild-type patients could be a cause of discrepancy between these two studies as *USP8* and *TP53* mutations are mutually exclusive. One *TP53* mutation was also found in corticotroph tumor in a study by Neou et al. who included 35 corticotroph PitNETs in their comprehensive molecular profiling (10). Thus, they reported similar frequency of *TP53* mutation to that observed in our study.

Characteristics of the reported *TP53*-mutated corticotroph tumors and clinical outcomes of the mutation carriers indicate that these mutations are related to adverse prognosis as it is observed in many human cancers (44). This was clearly shown by recent results by Perez−Rivas LG et al. who demonstrated significantly shorter overall survival in patients with *TP53*-mutated corticotroph PitNETs (42). Accordingly, exome-sequencing study by Sbiera S. et al. showed *TP53* variants in corticotroph macroadenomas (7) and Uzilov et al. found mutations in tumors with higher chromosomal instability (43). Formerly, *TP53* mutations were occasionally reported in invasive corticotroph adenoma (45) and ACTH-secreting pituitary carcinoma (46, 47). Recently they were also reported in 3 aggressive corticotroph tumors and 2 corticotroph carcinomas (48) as well as in corticotroph PitNETs with very high proliferation index (49). Similarly, we found *TP53* mutations in macroadenomas that are commonly characterized by invasive growth (excepting 1 of mutated tumors), and the overall survival of two CD patients with *TP53* mutations was below three years (one of the patients died from CD-related cause while the other due to COVID-19). Both our univariate and multivariate analyses of survival also indicate that the mutations are related to poor patients outcomes. Actually, the scarcity of the data (only 9 completed observations in survival analysis) and low frequency of *TP53* mutations prompt us to interpret this result cautiously. Fortunately, survival of patients with CD is relatively long in general, therefore, it is difficult to have completed observations in the survival analysis. We consider the follow up of 76.5 months in our study as relatively long, but further follow-up will improve the data. Nevertheless, when our results are interpreted in the context of previously published data revealing worse outcome in *TP53* mutated CD patients and the cases of *TP53* mutations in aggressive corticotroph PitNETs, it can be inferred that the mutations are related to poor prognosis.

Tumors that were screened for *TP53* mutations were also subjected to semi-quantitative assessment of TP53 expression with the use of antibody that is commonly applied for diagnostic purpose (clone DO-7). According to general opinion, *TP53*-mutated tumors are expected to be strongly immunoreactive due to a different half-life/turnover of mutated and wild-type protein (37). Indeed, we observed significantly higher TP53 immunoreactivity in tumors with missense *TP53* mutation as compared to wild-type tumors that presented mostly low protein expression. Importantly, a notable proportion of *TP53* wild-type tumors showed moderate TP53 expression indicating limited specificity of immunohistochemistry as a method for mutation testing in corticotroph PitNETs. The tumor with *TP53* frameshift mutation had very low protein expression. This result is in line with the general observation that most of TP53 protein-truncating mutations in cancer cause proteasomal degradation of defective protein (37). In contrast to effect of missense mutations, the presence of *TP53* frameshift variants in cancer is commonly related to loss of protein expression in IHC (37).

In summary, it is worth to note that among the published studies our research included one of the largest cohorts of unpreselected corticotroph PitNETs for mutational screening in the genes that, according to preliminary exome-seq data, were considered as tumor drivers. Our results suggest the favorable prognostic value of mutations in protein deubiquitinases genes in patients with CD. Confirmation of high prevalence of *USP8* mutations is also important since recent data indicated their predictive value for pharmacological treatment of CD patients with pasireotide (11–13).

The relevance of these mutations in silent tumors remains unknown due to a very low frequency. In contrary to *USP8* hot spot changes, *TP53* mutations are rare, occur in both functioning and silent tumors and appear to be associated with adverse clinical outcome in CD.

## Data availability statement

The datasets presented in this study can be found in online repositories. Variant call files generated with Ion Torrent Server software (Torrent Suite version 5.12.3) were deposited at the public repository Zenodo.org, DOI: https://doi.org/10.5281/zenodo.10680442.

## Ethics statement

The studies involving humans were approved by Ethics Committee of Maria Sklodowska-Curie National Research Institute of Oncology, Warsaw, Poland. The studies were conducted in accordance with the local legislation and institutional requirements. The human samples used in this study were acquired from a by- product of routine care or industry. Written informed consent for participation was not required from the participants or the participants’ legal guardians/next of kin in accordance with the national legislation and institutional requirements.

## Author contributions

MP: Conceptualization, Formal analysis, Funding acquisition, Investigation, Writing – review & editing. MS: Conceptualization, Data curation, Formal analysis, Investigation, Methodology, Writing – review & editing. PK: Investigation, Writing – original draft. NR: Investigation, Writing – review & editing. BM: Investigation, Writing – review & editing. SB: Data curation, Formal analysis, Conceptualization, Visualization, Writing – review & editing. AK: Conceptualization, Data curation, Formal analysis, Writing – review & editing. MM: Conceptualization, Data curation, Resources, Writing – review & editing. GZ: Data curation, Resources, Writing – review & editing. JK: Data curation, Resources, Writing – review & editing. PW: Data curation, Resources, Writing – review & editing. MB: Conceptualization, Formal analysis, Project administration, Writing – original draft.
