# Supplementary material for: Relevance of mutations in protein deubiquitinases genes and TP53 in corticotroph pituitary tumors
Source: Front Endocrinol (Lausanne). 2024 Feb 29;15:1302667. doi: 10.3389/fendo.2024.1302667 (PMC10937451; doi:10.3389/fendo.2024.1302667)
Supplement: Supplementary file 1 [file Table_1.docx]

Supplementary Table 1. Summary of *USP8*, *USP48*, *BRAF* and *TP53* mutational screening in corticotroph PitNETs.

| Gene | Identified variant | mutations count in CD patients | mutations count in silent corticotroph PitNETs |
| --- | --- | --- | --- |
| *USP8* |  | 41/100 (41%) | 4/47 (8.5%) |
|  | p.P720R | 22/100 (22%) | 0/47 |
|  | p.S718del | 11/100 (11%) | 3/47 (6.4%) |
|  | p.S718P | 6/100 (6%) | 1/47 (2.1%) |
|  | p.P720Q | 2/100 (6%) | 0/47 |
| *USP48* |  | 6/100 (6%) | 0/47 |
|  | p.M415V | 5/100 (6%) | 0/47 |
|  | p.M415I | 1/100 (6%) | 0/47 |
| *BRAF* |  | 0/100 | 0/47 |
| *TP53* |  | 2/81 (2.5%) | 2/47 (4.25%) |
|  | p.R181H | 1/81 (1.2%) | 0/47 |
|  | p.L111P | 1/81 (1.2%) | 0/47 |
|  | p.R273C | 0/81 | 1/47 (2.1%) |
|  | p.K382AsnfsTer40 | 0/81 | 1/47 (2.1%) |
